# Ovarian Aging-Like Phenotype in the Hyperandrogenism-Induced Murine Model of Polycystic Ovary

**DOI:** 10.1155/2014/948951

**Published:** 2014-02-19

**Authors:** Mohammad Amin Rezvanfar, Habib A. Shojaei Saadi, Maziar Gooshe, Amir Hosein Abdolghaffari, Maryam Baeeri, Mohammad Abdollahi

**Affiliations:** ^1^Department of Toxicology and Pharmacology, Faculty of Pharmacy, Tehran University of Medical Sciences, Tehran 1417614411, Iran; ^2^Pharmaceutical Sciences Research Center, Tehran University of Medical Sciences, Tehran 1417614411, Iran; ^3^Centre de Recherche en Biologie de la Reproduction, Université Laval, Québec City, QC, Canada G1V 0A6; ^4^Faculty of Medicine, Tehran University of Medical Sciences, Tehran 1417614411, Iran; ^5^Pharmacology and Applied Medicine Department of Medicinal Plants Research Center, Institute of Medicinal Plants, ACECR, Karaj, Alborz 141554364, Iran; ^6^International Campus, Tehran University of Medical Sciences (ICTUMS), Tehran 1417653861, Iran

## Abstract

There are prominently similar symptoms, effectors, and commonalities in the majority of characteristics between ovarian aging and polycystic ovarian syndrome (PCOS). Despite the approved role of oxidative stress in the pathogenesis of PCOS and aging, to our knowledge, the link between the PCO(S) and aging has not been investigated yet. In this study we investigated the possible exhibition of ovarian aging phenotype in murine model of PCO induced by daily oral administration of letrozole (1 mg/kg body weight) for 21 consecutive days in the female Wistar rats. Hyperandrogenization showed irregular cycles and histopathological characteristics of PCO which was associated with a significant increase in lipid peroxidation (LPO) and reactive oxygen species (ROS) and decrease in total antioxidant capacity (TAC) in serum and ovary. Moreover, serum testosterone, insulin and tumor necrosis factor-alpha (TNF-**α**) levels, and ovarian matrix metalloproteinase-2 (MMP-2) were increased in PCO rats compared with healthy controls, while estradiol and progesterone diminished. Almost all of these findings are interestingly found to be common with the characteristics identified with (ovarian) aging showing that hyperandrogenism-induced PCO in rat is associated with ovarian aging-like phenotypes. To our knowledge, this is the first report that provides evidence regarding the phenomenon of aging in PCO.

## 1. Introduction

Nowadays, couples considerably postpone childbearing to their late reproductive-age period due to increasing socioeconomic demands. Besides, industrialization of communities has added a wide variety of environmental risk factors that may lead to disorders, diseases, and cancers in various organs.

It has been shown that exposure to some environmental factors and chemicals during life may accelerate progression towards the end of functional reproductive period [1]. Accordingly, several environmental factors have been confirmed to contribute to reproductive aging [2]. Female reproductive system is unique in the fact that it displays physiologically much faster rate of aging and when it reaches a senescent state, other organs in the body remain generally healthy.

Ovary is one of the most important organs in the female reproductive system as well as an endocrine organ that undergoes aging by a continuous decreasing in the number of follicles, diminished quality of oocytes, menstrual irregularities, ovarian hormonal deficiency, anovulation, and subfertility leading to menopause which is the final step in this process [3].

Several mechanisms seem involved in aging which oxidative stress (OS) is considered as one of the most important ones [4]. OS occurs due to excessive formation of oxygen-derived and/or nitrogen-derived toxic products in the presence of minimal antioxidant activity [5].

At moderate concentrations, reactive oxygen species (ROS) regulate physiologic functions in female reproduction such as folliculogenesis, oocyte maturation, steroidogenesis, corpus luteal function, and luteolysis [5]. However, increased OS during ovarian aging may contribute to follicular atresia and diminishing quantity and quality of oocytes [6]. Substantial theories explain that a long lasting status of overproduced toxic free radicals, insufficient antioxidants, and exposure to endocrine disrupting chemicals may be involved in reproductive disorders which eventually confer acceleration of gonads senescence and premature reproductive aging.

Polycystic ovarian syndrome (PCOS) as an inflammatory condition is the leading cause of anovulatory infertility in reproductive-aged women. It is characterized by increasing androgen secretion, menstrual irregularity, oligo-ovulation/anovulation, polycystic ovaries (PCO), infertility, and pregnancy complications [7]. An ovarian cyst is developed as a result of hormonal imbalance and has an atretic fluid-filled follicular structure with thin granulosa cell walls [8]. Not only PCOS patients display reproductive features, but also they develop several metabolic risks of type 2 diabetes and cardiovascular diseases including abdominal obesity, insulin resistance (IR), hyperinsulinemia, glucose intolerance, hypertension, and metabolic syndrome [9]. There are several different diagnostic criteria for PCOS. Hence, regardless of ethnicity, PCOS has different prevalence in women at their functional reproductive period. According to Rotterdam criteria, it is estimated that the average prevalence of PCOS is approximately 18% that is too high [10].

Because of its complex pathogenesis and unrecognized etiology, no preventive measure has been implemented for PCOS to date. The roles of OS and chronic low-grade inflammation in the pathogenesis of PCOS and the potential benefits of antioxidants have been the subjects of recent studies [11, 12]. High levels of androgen have been proposed as an initial step in the majority of PCOS cases. Hyperandrogenism impairs maturation of developing follicles in ovaries and consequently leads to developing cystogenesis [13]. Extensive animal experimentations have indicated that prepubertal or pubertal exposure of low doses of androgens via aromatization to estrogen results in long-term reproductive consequences including constant estrous cycles, hyposteroidogenesis, anovulation, and development of cystic follicles at adulthood [14]. Moreover, exposure to exogenous estrogen in adulthood has been determined by having deleterious effects on the ovarian physiology and endocrinology which may ultimately lead to cystogenesis, loss of follicle pool, and early senescence [15]. There are some evidences implying that there might be an association between aging and PCO. For instance, it has been recently shown that accelerated aging in mouse induced by D-galactose was concurrent with ovarian cystogenesis [16]. However, the causal link between hyperandrogenism and accelerated ovarian aging is not definitely recognized. It is therefore essential to perceive what hormonal, cellular, molecular, metabolic, environmental, and/or genetic factors contribute to this condition and how they affect ovarian follicle development. We have recently documented the potential role of oxidative/nitrosative stress and inflammatory responses in the pathogenesis of hyperandrogenism-induced PCO in rat [17, 18]. However, negligible information exists on the hyperandrogenization effects on ovarian aging and the molecular basis of disturbed folliculogenesis and cystogenesis.

Overall, it should be of considerable interest to investigate possible development of ovarian aging in hyperandrogenic PCO rats. Therefore, the main objective of this study was to investigate the association of ovarian aging with hyperandrogenism-induced PCO through cellular, molecular, histopathological, biochemical, and endocrinal evaluations.

## 2. Materials and Methods

### 2.1. Reagents

Unless otherwise stated, all chemicals were purchased from Sigma-Aldrich (USA). Krebs-ringer-bicarbonate (KRB) and ethyl acetate, from Fluka (USA), rat TNF-**α** ELISA kit from Bender Med System (Austria), rat MMP-2 ELISA kitfrom Cusabio Biotech Co., Ltd, (China), steroid hormone radioimmunoassay kits from Neogen (USA), and letrozole from SOHA Pharmaceutical Co. (Iran) were used in this study.

### 2.2. Rats

Adult female albino *Wistar* rats (180–200 g) with normal estrous cycle were used in the study. Animals were randomly divided into two groups containing 10 rats each. All rats had *ad libitum* access to pelleted food and tap water and housed under controlled temperature (22–25°C) with a relative humidity of 40–55% and 12 h lights and dark cycle. Only females with at least three consecutive 4-5 d regular estrous cycles were used in the experiment. Throughout the entire treatment, animals were weighed and a vaginal smear was done daily (to determine the stage of the reproductive cycle) up to the day of autopsy. All rats received care in accordance with the national health guidelines and the study protocol was approved by Tehran University of Medical Sciences (TUMS) review board.

### 2.3. Sexual Cycle

To study whether treatments altered the estrous cycle, smears were obtained daily by vaginal washing and evaluated microscopically during the treatment period. As determined in previous studies [17, 18] the observation of cornified cells in the smears during a minimum of 10 consecutive days was defined as persistent estrous, indicating anovulation and development of follicular cysts. At the beginning of the experiments, all rats had regular cycles.

### 2.4. Treatments

The Control group of ten rats received only vehicle (0.9% NaCl solution) orally, once daily. The treatment group of ten rats was gavaged with letrozole once daily at a concentration of 1 mg/kg orally dissolved in 0.9% NaCl. Effective dose of letrozole (SOHA Pharmaceutical Co. Tehran, Iran) were selected upon previous experiments. The treatment period was 21 days [17, 18].

### 2.5. Sampling

After the last treatment, all rats weighed and anesthetized with ether and blood samples were directly taken from the heart. Blood samples were centrifuged at 1000 ×g for 15 min and collected sera stored at −70°C until assayed for sex steroids (estradiol, progesterone, and testosterone), as well as insulin, TNF-*α*, and OS markers. All rats were killed after anesthesia and freshly dissected ovaries were weighed and divided as follows: five of each group was immediately fixed in 4% (w/v) paraformaldehyde for histopathology, whereas the remaining tissues were immediately frozen at −70°C until used for determination of matrix metalloproteinase 2 (MMP-2) and oxidant-antioxidant markers.

### 2.6. Histopathological Studies

Five ovaries from each group were processed step by step through formalin fixation, paraffin embedding, and longitudinally and serially sectioned at 4 *μ*m with a rotary microtome placed on a glass slide, stained with H&E (Hematoxylin and Eosin), and assessed microscopically by two persons blinded to the origin of the sections. Only the follicles containing an oocyte nucleus were counted in every ovarian section. Follicles were characteristically divided to primordial follicles (an oocyte surrounded by a single layer of granulosa cells and a diameter <100 *μ*m), growing follicles (an oocyte surrounded by several layers of granulosa cells, without an antrum, and diameter of 100 to 300 *μ*m) or graafian follicles (a peripheral oocyte surrounded by cumulus cells and several layers of granulosa cells, an antrum, and diameter >300 *μ*m). Atretic follicles were characterized by scattered pyknotic nuclei in the granulosa cell layer, detachment of the granulosa cell layers, loss of oocyte-granulosa cell communication, fragmentation and malformation of the oocyte, disruption of the zona pellucida (ZP), and presence of cellular debris in the antrum of the follicle. Histopathological changes were also categorized from (−) to (+++) according to their severity, where (−) was not a prominent or obvious pathological finding, and scores of (+), (++), and (+++) represented pathological findings of <33%, <33–66%, and >66% of the atretic follicles, respectively. The average value of histopathological findings was taken into consideration and when there was disagreement between the two observers as to the interpretation of some histologic characteristics, the case was reexamined and conclusion was made by consensus.

### 2.7. Evaluation of Oxidative Stress Biomarkers

To assess the effect of hyperandrogenization on ovarian oxidant-antioxidant balance, the LPO index, ROS, and level of TAC were analyzed.

#### 2.7.1. Determination of Cellular Lipid Peroxidation (LPO)

The cellular LPO was assessed in blood and ovaries using thiobarbituric acid reactive substances (TBARS) assay as described in our previous work [19]. The LPO levels were expressed as the extent of malondialdehyde (MDA) production during an acid-heating reaction. Data were reported as *μ*g/mg of protein.

#### 2.7.2. Determination of Reactive Oxygen Species (ROS)

The isolated ovaries were placed in a mitochondrial isolation buffer containing 0.25 M sucrose, 20 mM KCl, 1.0 mM EDTA, and 5.0 mM HEPES (pH 7.4) at a weight: volume ratio of 1 : 10. Tissues were minced thoroughly and homogenized with a manual glass homogenizer at 0–4°C. A portion of the homogenate and blood serum were used to determine ROS production which was measured by use of fluorescence DCFH with some modifications as set up in our lab. The assay buffer contained 130 mM KCl, 5 mM MgCl_2_, 20 mM NaH_2_PO_4_, 20 mM Tris-HCl, 0.1 mM FeCl_3_, 1.7 mM ADP, and 0.1 mM NADPH and 30 mM glucose (pH 7.4) with a total volume of 200 *μ*L. Assay buffer contained 5 *μ*M DCFH-diacetate (DCFH-DA) dissolved in 1.25 mM methanol with 5 *μ*M final methanol concentration. For homogenates, 50 *μ*L (*∼*1 mg protein) was included in either assay medium and incubated at 37°C for 15 min. This permitted DCFH-DA to be broken by intracellular esterase to derive free DCFH. The rate of oxidation from DCFH to dichlorofluorescein (DCF) indicative of oxidant production was followed at the excitation wavelength of 488 nm and emission wavelength of 525 nm and measured every 6 min for 60 min using an ELISA F-2000 fluorescence spectrometer. The rate was linear for at least 60 min at various concentrations of protein present, corrected for the autooxidation rate of DCFH [20].

#### 2.7.3. Total Ferric Reducing Antioxidant Power Assay

Total antioxidant power of the ovaries and sera was evaluated by measuring the ability to reduce Fe^3+^ to Fe^2+^. To do that we used TPTZ which its interaction with Fe^2+^ results in the formation of a blue color, with a maximum absorbance at 593 nm. Data were expressed as mmol/L ferric ions reduced to ferrous per mg of protein, as described in our previous work [21].

### 2.8. Evaluation of Serum Insulin as a Metabolic Biomarker

After 8 h fasting, serum insulin concentration was analyzed by enzyme-linked immunoassay (ELISA) technique as described previously [20].

### 2.9. Evaluation of Inflammatory Biomarkers

Concentrations of TNF-**α** were assessed using a rat sandwich ELISA kit and expressed as pg/mg protein. According to the procedure, a color product is formed in proportion to the amount of cytokine present in the sample. After adding stop solution to terminate the reaction, absorbance was measured at 450 nm as the primary wavelength and 620 nm as the reference wavelength [22]. To determine the concentrations of TNF-**α** per unit of protein, the Bradford method was used to measure protein content using concentrated Comassie blue as reagent and BSA as the standard.

### 2.10. Evaluation of Matrix Metalloproteinase 2/Gelatinase A (MMP-2)

Ovarian samples, which had been preserved at −80°C, were warmed to −20°C and weighed. A phosphate buffer (pH 7.4), which was prepared in weight-appropriate quantities, was diluted 10 times. The tissue samples were homogenized, and then centrifuged in a refrigerated centrifuge at 2000 ×g for 15 minutes, and their supernatants were transferred into microtubes. The MMP-2 was analyzed using commercial quantitative immunoassay kit; (Cusabio Biotech Co., Newark, NJ, USA). Assay was conducted according to manufacturer's guidelines.

### 2.11. Evaluation of Sex Steroids

Ovarian steroidogenesis function after induction of ovarian cystogenesis was determined by competitive radioimmunoassay, using commercial RIA kits (Neogen, USA) as described in our previous studies [18, 19].

### 2.12. Statistical Analysis

Analysis of variance was used when several parameters of the two groups were compared. Differences between control and PCO group were calculated by Student's *t*-test. All analyses were conducted using StatsDirect 3.0.97. A *P* < 0.05 was considered as statistically significant. Results are presented as means ± SD.

## 3. Results

### 3.1. Body and Ovaries Weight (g)

The results showed that the hyperandrogenized rats gained significantly (*P* < 0.001) more body and ovarian weights in comparison to the controls (Table 1).

### 3.2. Sexual Cycle

With respect to the sexual cycle, all control rats showed regular estrous cycles with an expected time of 4-5 days between two consecutive cycles. However, all hyperandrogenized rats were completely acyclic and exhibited constant estrous.

### 3.3. Morphological and Histopathological Findings

**Figure 1:**
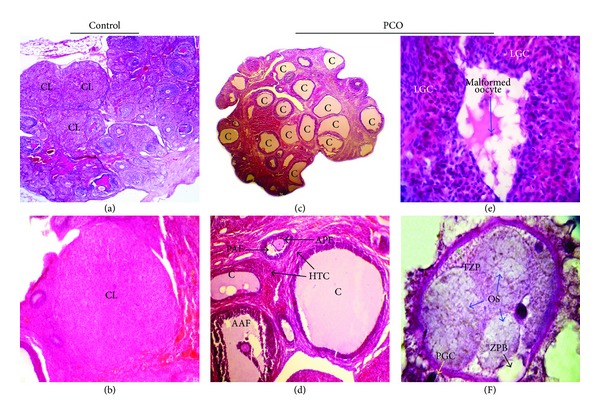
(a) Lower magnification (40x) of ovarian section showing normal folliculogenesis as well as several CL from the control rat. (b) Higher magnification (400x) of control ovary showing fresh CL. (c) Section of ovary from PCO rats showing multiple fluid-filled subcapsular cysts (40x). (d) Another section of ovary from PCO rats with two preantral and antral cystic follicles having hyperplastic theca cells along with thin granulosa cell layers. Two preantral and antral degenerating atretic follicles are also seen in this section (100x). (e) The completely luteinized granulosa cells are clear in an atretic follicle with a malformed oocyte (400x). (f) Higher magnification of a segmented oocyte in an atretic follicle with typical ZP thickness and also ZP breakdown and fragmentation (1000x). AAF: atretic antral follicle; APF: atretic preantral follicle; PAF: precocious antrum formation; C: cystic follicle; CL: corpora lutea; DGC: degenerated granulosa cells; HTC: hyperplasia of theca cells; LGC: luteinized granulosa cells; MO: malformed oocyte; OS: oocyte segmentation; PAF: precocious antrum formation; TZP: thicken zona pellucida; ZPB: zona pellucida breakdown.

Histological characteristics of the ovaries are shown in Tables 1 and 2. All ovaries of control females had corpora lutea (CL) confirming normal ovulation and contained follicles in various stages of development, including primary and secondary follicles, graafian follicles, and recently formed CL (Figures 1(a) and 1(b)). In the hyperandrogenized females, a clear absence of both large secondary and tertiary follicles as well as CL was evident and multiple follicular cysts were visible as fluid-filled sacs on the ovarian surface forming PCO (Tables 1 and 2; Figure 1(c)). Furthermore, the number of atretic preantral and antral follicles increased and ovarian theca-interstitial tissues are hyperplastic in hyperandrogenized ovary (Table 2 and Figure 1(d)). In addition, granulosa cells in the majority of atretic antral follicles were found luteinized (Figure 1(e)) and many of oocytes were malformed with the signs of zona pellucida (ZP) fragmentation in the atretic follicles (Figures 1(e) and 1(f)).

### 3.4. OS Biomarkers

In ovarian tissues and serum, LPO and ROS were significantly higher in the PCO group than in the controls (Table 3). Moreover, the ovarian and serum antioxidants activities were decreased significantly (*P* < 0.001) in the PCO rats compared to the controls.

### 3.5. Inflammatory and Metabolic Biomarkers

The hyperandrogenized PCO rats showed a considerable increase (*P* < 0.001) in serum TNF-*α* concentrations compared to the controls (68.2 ± 2.57 versus 41.9 ± 0.42, resp.). The serum insulin concentration was notably (*P* < 0.001) increased after hyperandrogenization in PCO rats as compared with the controls (1.1 ± 0.14 versus 0.32 ± 0.047; Table 3).

### 3.6. MMP-2 Activity

The mean tissue levels of MMP-2 in the ovaries are presented in Table 3. Statistically significant difference (*P* < 0.001) was found between the MMP-2 values of control and hyperandrogenized rats (23.9 ± 6 versus 65.5 ± 15.57, resp.).

### 3.7. Sex Steroid Concentrations

As seen in Table 4, hyperandrogenization significantly increased serum testosterone concentrations compared with the controls (*P* < 0.001, 80.22 ± 11.02 versus 21.58 ± 2.23, resp.). Meanwhile, serum concentrations of estradiol (*P* < 0.001, 12.42 ± 1.14 versus 60 ± 3.76) and progesterone (*P* < 0.001, 11.86 ± 1.36 versus 29.34 ± 3.13) were significantly reduced in the hyperandrogenized rats in comparison with the controls.

## 4. Discussion

Aging and reproduction biology are two rapidly growing fields of modern biomedical researches. Aging process is of interest not only to scientists but also to public health; hence, it has been the research question of many studies, including those in the field of reproductive biology. However, the association of aging with reproductive disorders has not been well studied and documented. In the present study we investigated the evidences of the ovarian aging characteristics with PCO as one of the most prevalent female reproductive disorder.

In this study we showed that treatment with letrozole-induced hyperandrogenism due to blocking the conversion of androgens to estradiol which leads to higher serum testosterone concentrations (hyperandrogenemia). Hyperandrogenemia was associated with ovarian cystogenesis and significantly increased LPO and total ROS (markers of OS) and decreased TAC (marker of antioxidant potential) in PCO rats. In addition, synthesis or release of inflammatory mediators like TNF-**α** and concentrations of insulin and MMP-2 activity were significantly increased by hyperandrogenization. These molecular and biochemical alterations were consistent with histological evidence of significant disruption in microscopic characters of folliculogenesis when compared to the control group. Histopathology revealed a large increase in the number of atretic and cystic follicles, lack of CL formation, anovulation, and sexual acyclicity in hyperandrogenized rats which was due to aberrant folliculogenesis. Increased ovarian weight seemed to be a result of increased number of fluid-filled cysts and large atretic follicles within ovary of PCO cases. Almost all of these findings were interestingly found to be common with the characteristics identified with (ovarian) aging as discussed. Interestingly, we observed that there was a close association between identified symptoms and effectors regarding ovarian aging in hyperandrogenized PCO rats so that all of these characteristics have been related to (ovarian) aging in several recent studies [3, 4, 6, 7, 16].

### 4.1. Low Estradiol Levels and High Concentrations of Androgen (Hyperandrogenemia) Leading to Accelerated Follicular Atresia

Letrozole blocks aromatization of testosterone to estradiol leading to a significant reduction in serum estradiol concentration and consequently accumulation of nonaromatizable androgens [17, 18]. Furthermore, due to the developing of CL or decreased number of mature CL as a consequence of anovulation, decreased concentration of serum progesterone was predictable in PCO cases. These hormonal disturbances are involved in constant estrous manifestation. Estradiol is known to play important roles in preventing OS so that lower estradiol concentrations are associated with an increase in follicular OS [23]. A meaningful depletion in estradiol levels causes apoptosis and oxidative DNA damage. As well, elevated OS resulting from reduced estradiol concentrations would predispose granulosa cells to apoptosis in preovulatory follicles [24]. Therefore, it is likely that the decreased levels of estradiol, at least in part, stimulates progressive follicular atresia and predisposes the ovary to be senescent prematurely.

### 4.2. High Concentrations of Insulin (Hyperinsulinemia) Leading to Hyperandrogenemia and Accelerated Follicular Atresia

PCOS is associated with hyperinsulinemia. Hyperinsulinemia has been determined to stimulate the ovarian cystogenesis causing an increase in the size of the cystic follicles and in the size of the ovary [25]. Insulin excess stimulates androgen production by theca cells and elevates serum free testosterone levels, thereby perpetuating ovarian hyperandrogenism [26]. In the present study, ovarian theca-interstitial cell growth was consistent with high concentrations of testosterone and insulin in comparison with controls. Ovarian mesenchyma (theca-interstitial tissues) are a major source of androgens and normal ovarian function, including folliculogenesis and steroidogenesis, which requires effective mechanisms regulating theca-interstitial growth and function. However, in PCOS, ovarian theca-interstitial tissues are hyperplastic due to increased cellular proliferation and/or reduced apoptosis which is concomitant with androgens oversynthesis [27]. In this regard, androgens might enhance apoptosis of granulosa cells leading to increased rate of follicular atresia [28].

### 4.3. Increased TNF-*α* Activity Leading to Sustained Inflammatory Status, Progressive Ovarian Cystogenesis, and Follicular Atresia

In the present study, PCO rats had higher serum TNF-*α* concentrations than controls. There is a positive relationship between hyperandrogenism and serum TNF-*α*  excessive concentration as hallmarks in patients with PCOS [11, 12]. TNF-*α* is considered as an initiator of the inflammatory cascade. Furthermore, higher TNF-*α* production is involved in OS [14, 29] which arrests follicular development [30] and correlates with poor oocyte quality [31]. A similar situation has been determined in female rats, where the levels of TNF-**α** and LPO were elevated in old ovariectomized animals as an experimental model of aging [32]. Therefore, in hyperandrogenism condition the crosstalk between OS and proinflammatory cytokines, particularly, TNF-*α* seem to play a pivotal role in the progression of ovarian cystogenesis, follicular atresia and premature ovarian dysfunction as was evident in the present study.

With respect to aging and TNF-**α**, age-dependent increase in TNF-**α** was found in senescence-accelerated mice [33]. It has been shown that aging is mechanistically associated with the gene expression of pro-inflammatory cytokines such as TNF-*α*, leading to inflammation and apoptosis [34]. Moreover, it has been well determined that TNF-**α** is able to markedly increase cellular senescence through stimulation of prolonged inflammation [35].

### 4.4. Overproduction of Intraovarian Toxic Free Radicals along with a Depletion of TAC

The role of ROS in the pathogenesis of PCOS has been the subject of numerous studies and emerging evidences that show that both ROS and the activation of inflammatory pathways play a central role in PCOS [5, 11, 12]. In the present study, we showed that hyperandrogenization significantly increased LPO and total ROS (markers of OS) and decreased TAC (marker of antioxidant potential) in PCO rats. Aging has been shown to be mechanistically associated with impaired mitochondrial function which is related to toxic free radicals overproduction and decreased antioxidant activity [36].

### 4.5. The Alternation in Extracellular Matrix (ECM)

One of the main findings reported in this study is that hyperandrogenization increased MMP-2 activation in rats with respect to controls. Matrix metalloproteinases (MMPs) which are a group of matrix-degrading enzymes are involved in the breakdown of all kinds of ECM proteins and so play an important role in various physiological processes as well as pathological states of disease. It has been exhibited that follicular rupture requires focal degradation of the apical ECM that is controlled, in part, by ovarian MMPs. The MMP-2 has been proposed to play an important role in separation of the granulosa cells from the theca cells [37]. Increased level of MMP-2 in our experiment was in agreement with a previous report showing elevated MMP-2 levels in obese women with PCOS [38]. The imbalances in circulating MMPs are associated with excess circulating androgens in PCOS women [39]. In this regard, MMP-2 expression has been shown to be stimulated by androgen via androgen receptor transactivation [40]. There is also a correlation between MMPs and the quality of the developing follicles [41]. Moreover, luteinized granulosa cells (as an atresia characteristic) from women with PCOS have been approved to exhibit greater MMP-2 activity [41]. In terms of aging, the overexpression of several metalloproteinases in senescent cells has been reported [42]. Therefore, it is reasonable to speculate that MMP-2 may be associated with inappropriate follicular atresia in PCOS cases which eventually leads ovary to be senescent faster than the normal condition.

### 4.6. Ovarian Aging-Like Phenotype in the Hyperandrogenism-Induced PCO

Ovarian aging process is characterized by the disappearance of the preovulatory follicle pool in the ovary and ceases in the female fertility due to lack of CL formation, anovulation, and discontinued activity of sexual cycling [43]. It has been proved that progressive follicular atresia by apoptosis at any stage could accelerate follicular depletion from the ovary and result in premature reproductive senescence [44]. Furthermore, hyperandrogenization as one of the common models of PCO seems to exhibit some aging-specific characteristics. Supporting this subject, postnatal androgenization has been shown to induce accelerated aging of ovarian interstitial glands and transformation of large antral into cystic follicles as common similarities between hyperandrogenized and aging rats [45]. It is believed that menopause associated with various diseases is mediated through depression of estradiol levels [46]. Interestingly, condition of chronic estradiol deficiency and higher androgen levels (as seen in our results) would lead to many age-related changes like obesity, metabolic syndrome, diabetes, skeletal abnormalities, and cardiovascular disturbances [47] where all of these diseases and disorders can be observed in PCOS women [7]. Considering the facts and results of this study, it would not be surprising to consider that the PCO(S) and ovarian aging notably have similar symptoms, effectors, and commonality in majority of their characteristics. Although not investigated in this study, there are several findings which support this idea. For instance, recently in mouse model of accelerated aging, irregular estrous cycles and ovarian cysts, as common signs of ovarian failure, were identified [16]. Another good example is advanced glycation end products (AGEs). Accumulation of AGEs is well known among many causes of aging [48]. AGEs contribute directly to protein damage, toxic stress, and increasing the inflammatory reactions [49]. Recent studies have shown a clear connection between PCOS and increased AGEs in the body so that AGEs and its receptor were highly expressed in granulosa cells of the PCOS ovary [50]. The AGEs seem associated with anovulation and hyperandrogenism [51].

Altogether, according to above discussion, the first concept coming to mind is that during PCOS condition, constant exposure of oocytes and other ovarian cells to OS, and hormonal alternations predispose the ovarian follicles to progressive rate of atresia leading to exhausted ovaries with reduced number of intact follicles. Therefore, ovarian oocyte reserved will be washed up earlier in hyperandrogenism condition, in accordance with premature onset of acyclicity and constant estrous. Moreover, supraphysiologic long-lasting androgenic microenvironment may eventually affect process of follicular recruitment, growth and ovulation leading to early ovarian senescence [15] via disruption of intraovarian milieu. This is a phenomena resembling premature/accelerated ovarian aging. According to recent studies including our reports and based on current data, we give the idea that hyperandrogenism triggers ovarian senescence in PCOS cases through the following mechanisms: (1) pivotal decrease in estradiol levels and high concentrations of androgen leading to accelerated follicular atresia; (2) increased TNF-*α* activity leading to OS and sustained inflammatory status; (3) overproduction of intraovarian toxic free radicals along with a depletion of TAC.

Although, a close association with the symptoms and effectors of hyperandrogenemia-induced PCO and (ovarian) aging was observed in our study, a number of important questions may be raised: What is the mechanistically precise association of PCOS with ovarian aging? Is it logical to consider PCOS as one of the contributors of accelerated development of ovarian senescence in risky individuals? And in general, is PCOS a unique and sophisticated type of accelerated ovarian aging phenomenon? This is an idea/hypothesis which will be well scrutinized and discussed appropriately as a research question in our upcoming project.

## 5. Conclusion

To our knowledge, this is the first report that provides evidence on phenomenon of aging in PCO. Our results showed that PCO(S) which is coupled with premature onset of constant estrous, OS, and proinflammatory condition may lead to premature transition to reproductive senescence via endocrine disruption and develops as one of the features of accelerated aging at the level of the ovary. Further studies are required to clarify precise mechanisms explaining similarities of hyperandrogenism-induced PCO and ovarian aging.

## Figures and Tables

**Table 1:** Comparative assessment of weight and histopathological changes of ovary in experimental and control groups.

	Control	PCO
Body weight (g)	231.2 ± 1.11	249.8 ± 0.81***
Ovary weight (g)	0.044 ± 0.002	0.06 ± 0.002***
Luteinization of follicular wall and granulosa cells	—	+++
Vascularization of follicular wall	—	+++
Degenerated and deformed oocyte	—	+++
Pyknosis and chromatinization of granulosa cells	—	+++
Disintegration and dispersion of granulosa cells	—	+++
Hyperplasia of theca cells	—	+++
Breakdown and fragmentation of ZP	—	++

*represents a significant difference between control and PCO groups.

The symbols represent statistical significance: *P < 0.05, **P < 0.01, and ***P < 0.001.

Intensity of the histopathological changes: (—) no change; (+) slight change; (++); marked change; (+++) sever change.

**Table 2:** Comparison of mean number of atretic and cystic primordial, growing, and graafian follicles and corpus luteum in PCO and control groups.

	Control	PCO
Mean number of atretic primordial follicles	35.81 ± 1.34	52.27 ± 1.01***
Mean number of atretic growing follicles	52.45 ± 0.57	81.18 ± 0.91***
Mean number of atretic graafian follicles	8.72 ± 0.33	27.36 ± 0.38***
Mean number of cystic primordial follicles	0	0
Mean number of cystic growing follicles	0.18 ± 0.12	7.72 ± 0.38***
Mean number of cystic graafian follicles	0.82 ± 0.18	10.9 ± 0.56***
Mean number of corpus luteum (CL)	5.27 ± 0.19	0.36 ± 0.15***

*represents a significant difference between control and PCO group.

The symbols represent statistical significance: *P < 0.05, **P < 0.01, and ***P < 0.001.

**Table 3:** Comparative assessment of OS and inflammation parameters in blood and ovary of PCO and control groups.

	Control	PCO
Blood LPO (*μ*mol/mL)	4.09 ± 0.32	12.72 ± 0.27***
Ovary LPO (*μ*mol/mL)	3.45 ± 0.28	14.21 ± 0.56***
Blood ROS (nmol/min/mg protein)	0.78 ± 0.18	2.76 ± 0.32***
Ovary ROS (nmol/min/mg protein)	0.04 ± 0.00	0.14 ± 0.01***
Blood TAC (nmol/L per mg protein)	209.52 ± 4.66	116.60 ± 4.21***
Ovary TAC (u/mg)	7.68 ± 0.49	2.91 ± 0.16***
Serum TNF-*α* (pg/mg protein)	41.90 ± 0.42	68.20 ± 2.57***
MMP-2 (ng/mg protein)	23.90 ± 6.00	65.50 ± 15.57***
Insulin (ng/mL)	0.32 ± 0.05	1.10 ± 0.14***

*represents a significant difference between control and PCO group.

The symbols represent statistical significance: *P < 0.05, **P < 0.01, and ***P < 0.001.

**Table 4:** Sex hormone levels in PCO and control groups.

	Control	PCO
Testosterone (ng/dL)	21.58 ± 2.23	80.22 ± 11.02***
Progesterone (ng/mL)	29.34 ± 3.13	11.86 ± 1.36***
Estradiol (pg/mL)	60.00 ± 3.76	12.42 ± 1.14***

*represents a significant difference between control and PCO group.

The symbols represent statistical significance: *P < 0.05, **P < 0.01, and ***P < 0.001.
